# regioneReloaded: evaluating the association of multiple genomic region sets

**DOI:** 10.1093/bioinformatics/btad704

**Published:** 2023-11-21

**Authors:** Roberto Malinverni, David Corujo, Bernat Gel, Marcus Buschbeck

**Affiliations:** Program of Myeloid Neoplasms, Program of Applied Epigenetics, Josep Carreras Leukaemia Research Institute (IJC), Campus Can Ruti Site, Badalona 08916, Spain; Program of Myeloid Neoplasms, Program of Applied Epigenetics, Josep Carreras Leukaemia Research Institute (IJC), Campus Can Ruti Site, Badalona 08916, Spain; Germans Trias i Pujol Research Institute (IGTP), Can Ruti Campus, Badalona, Barcelona 08916, Spain; Program of Myeloid Neoplasms, Program of Applied Epigenetics, Josep Carreras Leukaemia Research Institute (IJC), Campus Can Ruti Site, Badalona 08916, Spain; Germans Trias i Pujol Research Institute (IGTP), Can Ruti Campus, Badalona, Barcelona 08916, Spain

## Abstract

**Motivation:**

Next-generation sequencing methods continue improving the annotation of genomes in part by determining the distribution of features such as epigenetic marks. Evaluating and interpreting the association between genomic regions and their features has become a common and challenging analysis in genomic and epigenomic studies.

**Results:**

With *regioneR* we provided an R package allowing to assess the statistical significance of pairwise associations between genomic region sets using permutation tests. We now present the R package *regioneReloaded* that builds upon *regioneR’s* statistical foundation and extends the functionality for the simultaneous analysis and visualization of the associations between multiple genomic region sets. Thus, we provide a novel discovery tool for the identification of significant associations that warrant to be tested for functional interdependence.

**Availability and implementation:**

*regioneReloaded* is an R package released under an Artistic-2.0 License. The source code and documentation are freely available through Bioconductor: http://www.bioconductor.org/packages/regioneReloaded.

## 1 Introduction

In genetic and epigenetic research, high-throughput sequencing techniques produce vast amounts of data, making the meaningful interpretation of multiple genomic datasets one of the biggest challenges for analysis. One of the most common ways in which data resulting from genomic and epigenomic analysis is represented are “region sets” that are a list of genomic regions defined by specific start and end coordinates in a reference genome. Frequently generated region sets include ChIP-Seq or CUT&RUN peaks, differentially methylated regions, interacting regions from chromosome conformation capture methods or regions with copy number alterations or mutations. Transcriptomic data can be converted into sets of regions by different means such as by extracting the promoters of differentially expressed genes. Moreover, features of the genomic sequence itself, such as annotated regulatory elements, repetitive elements, highly conserved regions or other motifs can be chosen as region sets for further analysis.

To interpret the meaning of overlaps between regions sets, in 2016 we published *regioneR*, an R package developed for the statistical evaluation of the association between sets of genomic regions (Gel *et al.* 2016). *RegioneR* is based on comparing the observed biological association with a random distribution obtained by permuting the original regions. To overcome some of *regioneR*’s limitations, we now present *regioneReloaded*, an R package that is the evolution of *regioneR* and allows to simultaneously calculate the statistical association between multiple regions sets. While Heger *et al.* (2013) introduced a comparable methodology with their Python command-line tool GAT, our R package, available in the Bioconductor repository, emphasizes permutation-based analyses for the association of multiple region sets with flexible randomization and evaluation methods. Importantly, we have developed *regioneReloaded* as a modular and customizable framework for genomic association studies, with built-in functionality but the option to include custom randomization and evaluation functions for complex biological scenarios or to potentially integrate methods from other packages.

**Figure 1.**
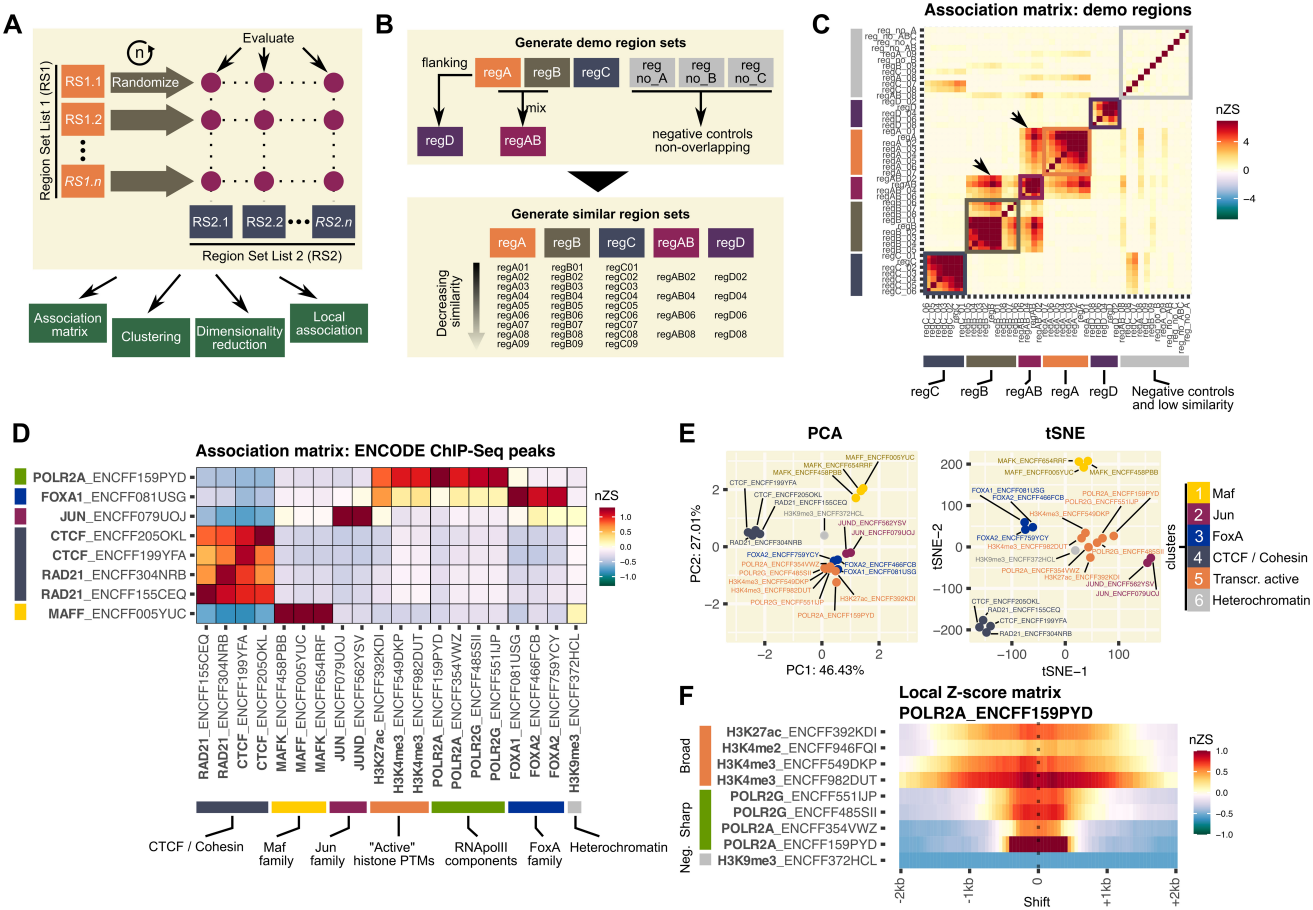
*regioneReloaded* allows to calculate and compare pairwise associations between multiple region sets. (A) Simplified scheme of the functionality of *regioneReloaded* using the randomization method of *regioneR* (Gel *et al.* 2016). A single randomization step is performed on each region set in RS1 by permuting the regions *n* times, and the association is evaluated repeatedly for each element in RS2. The resulting association results are stored in a matrix which can be clustered and visualized by different methods. In additional, the local nature of associations can be further investigated. (B) Scheme of how we have created an example dataset with demo region sets from an artificial AlienGenome. The artificial reference genome has 4 chromosomes of 0.1, 0.5, 1, and 2 Mb in size. Each region set has 100 regions of a mean of 100 bp ± SD of 50 bp. (C) Association matrix plot of normalized *z*-scores of the multiple permutation test on the region sets of the example dataset AlienGenome using *resampleGenome* as randomization function with 1000 permutations and *numOverlaps* as the evaluation function. Arrows point at the detected association between regAB, regA and regB region sets. (D) Association matrix of normal *z*-score values obtained after analyzing ChIP-Seq peaks of different chromatin-associated proteins and histone PTMs, annotated by the ENCODE project from data generated in the HepG2 cell line. The row and column names show the ID of the corresponding file in the ENCODE database, which in some cases contains experimental replicates. The multiple permutation test was performed using 1500 regions from the upper quartile of peaks with the higher score from each file, the evaluation function *numOverlaps*, 2000 permutations and the randomization function *resampleRegions*, with a universe made of merging all the regions in all files. Normalized *Z*-score values with an adjusted *P* value > 0.05 are displayed as 0. Colored boxes represent the biological annotation of the detected associated factors. (E) Dimensionality reduction plots can be generated directly from the association results and clustering. In the figure, PCA and tSNE plots showing the same data as in D and labeled as clustered by hierarchical clustering. (F) Matrix plot of the local normal *Z*-score profiles of one of the POLR2A samples against a subset of the region sets analyzed in D. Results were obtained using *numOverlaps* as an evaluation function, a window of 2 kb, a step of 50 bp, 2000 permutations and *resampleRegions* with a universe made of merging all the regions in all files. Colored boxes represent the annotation of signals that have a “broad” or “sharp” association profile.

*regioneR* performs pairwise association analysis providing *Z*-scores (ZS) as statistical value for the association. The ZS depends on the size of the input region sets and thus ZS of different pairwise analyses cannot be directly compared. In *regioneReloaded*, we introduce a normalized *Z*-Score to allow the direct comparison of *z*-scores from different tests. In addition, when scaling up the number of pairwise comparisons for larger datasets, the increase in calculation time becomes a serious limitation. To overcome this, a single randomization step per region set is performed followed by multiple evaluations of the association with other region sets, significantly cutting down computation time (Fig. 1A). Moreover, *regioneReloaded* includes an object class to conveniently contain all the results from multiple permutation tests and associated parameters of how it was performed, as well as functions to analyze clusters of associations to facilitate the interpretation of the results and plotting functions for data visualization and the generation of publication-ready graphs (Fig. 1A). While we will present here the core functionality of *regioneReloaded*, we point the reader to the *regioneR* application note and documentation for a thorough description of the permutation-based testing strategy of genomic associations (Gel *et al.* 2016).

## 2 The normalized *Z*-score for permutation tests

*regioneReloaded* uses the statistical framework previously developed in *regioneR* and allows to simultaneously calculate associations of different region sets. Values of ZS obtained in different tests depend on the number of regions present in the permuted region set (*n*). To make them comparable, we introduce the concept of the normalized *Z*-score (nZS) by dividing the ZS by the square root of *n*. This reduces the dependency of the nZS on *n*, which facilitates direct comparison of different pairwise associations with different sample sizes and, in addition, facilitates working with subsets of the data to reduce calculation times.

$$\mathrm{nZS}=ZS/\sqrt{n}$$

While the absolute value of ZS steadily increases as more regions from the dataset are included in the test, the nZS shows a lower dependency with the number of regions analyzed and stabilizes faster after including a portion of the dataset (Supplementary Fig. S1A). This shows that sampling a portion of large datasets is a viable strategy to reduce computation time without impacting the capacity to obtain meaningful nZS values. While we propose the nZS as a valuable tool for stabilizing ZS results from different analysis, *regioneReloaded* includes the option for the user to report all analysis, clustering and visualizations with the ZS instead to capture the unaltered statistical value of the permutation test.

## 3 Multiple permutation test

The *regioneReloaded* R package offers a significant improvement over its predecessor *regioneR* by efficiently conducting multiple parallel permutation analyses for all-against-all input region sets. This refers to the computation of permutation tests between all possible combinations of elements within two lists of region sets that we shall term RS1 and RS2. In a typical “symmetrical” analysis, RS1 and RS2 will contain the same number of region sets. The *crosswisePermTest* function performs this analysis and has been optimized for efficiency by utilizing a single randomization call to each element of RS1, followed by repeated evaluations of the association with all elements of RS2. Since randomization is the most computation-intensive step of the permutation test, this approach is effective at optimizing the total calculation time. *regioneReloaded* can use all the randomization and evaluation functions available in *regioneR* as well as custom functions. The resulting *P*-values are then adjusted to correct for multiple testing, using Benjamini-Hochberg as default but allowing other methods through the function arguments. The main outcome of this function is an S4 object of class *genomatriXeR* containing the results of the multiple permutation tests.

## 4 Creation of the example dataset: the AlienGenome

To showcase the functionality of *regioneReloaded*, we have created and included a small demo dataset allowing fast test calculations (Fig. 1B). Specifically, we have created an artificial genome called “AlienGenome” comprised of four chromosomes of 0.1–2 Mb in length. As starting point, we independently created three main region sets termed regA, regB, and regC by applying the *createRandomRegions* function from the *regioneR* package on the AlienGenome. Each region set includes 100 regions with an average width of 100 bp and a standard deviation of 50 bp. Next, we created two regions sets that are related to regA and regB. regAB shares half its regions with regA and half with regB. regD is comprised of regions within 300 bp distance of the regions of regA without overlapping, effectively containing “flanking” regions. For all these datasets, we have created region sets of varying degrees of similarity. Using the *similarRegionSet* function we created regions sets that share a percentage of similarity ranging from 90% to 10% for regA, regB, and regC and 80%–20% for regD and regAB. As negative controls, we created random region sets that do not contain any shared region of regA, regB, regC, or regAB using the *createRandomRegions* and *substractRegions* functions from *regioneR*.

## 5 Visualization and clustering

### 5.1 Association matrix visualization and clustering

The *regioneReloaded* package has several functions specifically designed to work with the association results contained in the *genomatriXeR* object, mainly focused on visualizing the association data and identifying association clusters. In particular, the *makeCrosswiseMatrix* function will create an array of nZS and cluster the resulting matrix automatically selecting the most efficient method from those available in *hclust* (alternatively, a specific method can be chosen). The matrix can be visualized using *plotCrosswiseMatrix* as an association matrix (nZS or ZS values) or as a correlation matrix with Pearson correlation of R values. By default, the matrix will be ordered using the clustering results, which can be retrieved with *getHClust* for further analysis. Clustering of region sets with similar association patterns is aimed at identifying potential functional interactions of biological significance. Using the example dataset, we can observe well-defined clustering of the predicted association between regA, regB, and regC sets and the region sets that are similar to them (Fig. 1C). As expected, regAB associates with both regA and regB, while control region sets show no association with any other region sets.

As an example of analysis of real biological data, we have performed multiple association testing of several ChIP-Seq peaks of chromatin associated proteins and histone post-translational modifications (PTMs), identified by the ENCODE project in the HepG2 cell line (Fig. 1D) (Dunham *et al.* 2013). Using *regioneReloaded*, we detect strong associations between described protein complexes, mainly: CTCF and cohesin complex member RAD21 (Rao *et al.* 2014); Maf, Jun, and FoxA family transcription factors (Bochkis *et al.* 2012, Kannan *et al.* 2012, Bejjani *et al.* 2019) and RNA polymerase II subunits POLR2A (RPB1) and POLR2G (RPB7) (Armache *et al.* 2005). Notably, we also identify association of histone PTMs H3K4me3 and H3K27Ac, enriched at actively transcribed promoters, with RNApolII subunits (Kundaje et al. 2015). On the contrary, the heterochromatic mark H3K9me3 shows a negative association with the aforementioned factors related to transcriptional activity. Notably, the methods provided in *regioneReloaded* are also suitable for analyzing associations with other genomic features beside ChIP-Seq enrichment sites, such as gene promoters, exons, or repetitive elements (e.g. Supplementary Fig. S1B).

### 5.2 Dimensionality reduction plots

Dimensionality reduction transformations allow the representation of complex multi-dimensional data in a low-density space while retaining the most meaningful properties of the original data. *regioneReloaded* provides the function *plotCrosswiseDimRed* for producing two-dimensional visualizations of the association matrix using three of the most widely used algorithms for this purpose: PCA, tSNE, and UMAP (Fig. 1E and Supplementary Fig. S1C). The *plotCrosswiseDimRed* function can be controlled with several parameters to aid in visually exploring the association and clustering of the data and to produce publication-ready plots with highlighted features of interest. Note that the clusters are obtained from the association matrix itself and not from the projected values on the reduced dimensions. Moreover, all plots produced by the package are *ggplot* objects that can be further customized.

## 6 Multiple local *Z*-score test

The local *Z*-score testing as implemented in *regioneR* is based on shifting the positions of the regions in a region set in defined steps and re-evaluating the observed association and corresponding ZS. This strategy is aimed at identifying the local nature of the association between two region sets. While a sharp decline in ZS after positional shifting indicates that the association is very dependent on the exact positions of the regions, a flat profile indicates a “regional” association where the ZS is maintained after shifting. An example for such an association is that of narrow transcription factor ChIP-seq peaks embedded in a broad region marked by a histone modification. Moreover, the local *Z*-score profile can reveal “flanking” associations in which the ZS is originally low but increases when the regions are shifted. Thus, the local *Z*-score can be a valuable tool to interpret the biological meaning and significance of the associations between region sets.

*regioneReloaded* includes the *multiLocalZScore* function to obtain multiple local Z-score profiles of one region set versus all the elements on a list of region sets. The results will be stored in an object of class *multiLocalZScore* and the matrix of local Z-scores can be computed and clustered by the *makeLZMatrix* function. The results can be finally visualized by *plotLocalZScoreMatrix* which produces a heatmap-type plot where each row is a 1D representation of the local Z-score profile resulting from the positional shifting of a region set before calculating the association with the reference region set.

Using the demo dataset, we can compute the profiles of all local associations in respect to regA. As shown in Supplementary Fig. S1D, regA is centrally associated with all its similar region sets and laterally with the flanking region set regD and its derivatives. When analyzing biological data from ENCODE, we observe a “sharp” association of POLR2A with a second RNApolII subunit, while the local *Z*-score profile is spread across 1–2 kb for its association with broad histone marks (Fig. 1F).

## 7 Conclusion

*regioneReloaded* is based on the permutation test based statistical framework provided by *regioneR* and implements an extension of the method for analyzing and visualizing simultaneously the association between multiple genomic region sets. We have kept the package modular and customizable to make possible the integration of current and new tools for genomic association testing while retaining a framework for computationally optimal multiple testing. For example, the *matchRanges* function from the *nullranges* package (Davis *et al.* 2023) could be used to either generate “control” region sets with matched covariates as input for the permutation test. We have put particular importance on functions that enable the identification but also the visualization of clusters of associated regions. As clusters are likely indicative of shared biological functions, our package can be used as a discovery tool. *regioneReloaded* is thus a new and valuable addition to the repertoire of available tools in the Bioconductor repository for the analysis and interpretation of high-content genomic datasets.

## Supplementary Material

btad704_Supplementary_Data

## Data Availability

The data underlying this article are available in the ENCODE Portal at https://www.encodeproject.org/, and can be accessed with the unique identifiers as described in the manuscript. The demo sample data is included as part of the R package available in Bioconductor at 10.18129/B9.bioc.regioneReloaded.
