# Supplementary material for: regioneReloaded: evaluating the association of multiple genomic region sets
Source: Bioinformatics. 2023 Nov 21;39(11):btad704. doi: 10.1093/bioinformatics/btad704 (PMC10681856; doi:10.1093/bioinformatics/btad704)
Supplement: btad704_Supplementary_Data [file btad704_supplementary_data.zip › Supplementary_figure_legends.docx]

**Supplementary figure 1**

**A.** After normalization, z-scores are much less dependent of the size of the region sets tested. Calculated z-scores (ZS) and normalized z-scores (nZS) are shown for the association of increasing subfractions of the indicated region sets from ENCODE in HepG2 cells. Association tests were performed with *randomizeRegions* as permutation function, *numOverlaps* as evaluation and 5000 permutations. The upper quartile of peaks with the higher score from each file was used as region set.

**B.** Association matrix of normal z-score values obtained analyzing the association of ChIP-Seq peaks annotated by the ENCODE project in HepG2 cells against a set of genomic features. The upper quartile of peaks with the higher score from each file was used as region set. Gene promoters, introns and exons where obtained from the TxDb.Hsapiens.UCSC.hg38.knownGene R package, while LINE1 repeat locations where extracted from the UCSC RepeatMasker annotations (Sep2021) for Human (hg38).

**C.** PCA is one of the three options for dimensionality reduction and shows the same data as in B with ellipses denoting the calculated clusters of regions with similar associations. Arrows point at the position of the main demo region sets.

**D.** Matrix plot of the local Z-score profiles of regA against the rest of the region sets in the AlienGenome (Figure 1B), using *resampleGenome* as randomization function with 100 permutations, *numOverlaps* *as* evaluation and position shifts of 10bp steps within a window of 600bp. Arrows point to the detected “flanking” association between regA and regD.
